# Genes Encoding Mammalian Oviductal Proteins Involved in Fertilization are Subjected to Gene Death and Positive Selection

**DOI:** 10.1007/s00239-018-9878-0

**Published:** 2018-11-20

**Authors:** Carla Moros-Nicolás, Sophie Fouchécourt, Ghylène Goudet, Philippe Monget

**Affiliations:** PRC, INRA, CNRS, IFCE, Université de Tours, 37380 Nouzilly, France

**Keywords:** Protein evolution, Oviduct, Fertilization, Pseudogene, Positive selection

## Abstract

Oviductal proteins play an important role in mammalian fertilization, as proteins from seminal fluid. However, in contrast with the latter, their phylogenetic evolution has been poorly studied. Our objective was to study in 16 mammals the evolution of 16 genes that encode oviductal proteins involved in at least one of the following steps: (1) sperm–oviduct interaction, (2) acrosome reaction, and/or (3) sperm–zona pellucida interaction. Most genes were present in all studied mammals. However, some genes were lost along the evolution of mammals and found as pseudogenes: annexin A5 (ANXA5) and deleted in malignant brain tumor 1 (DMBT1) in tarsier; oviductin (OVGP1) in megabat; and probably progestagen-associated endometrial protein (PAEP) in tarsier, mouse, rat, rabbit, dolphin, and megabat; prostaglandin D2 synthase (PTGDS) in microbat; and plasminogen (PLG) in megabat. Four genes [ANXA1, ANXA4, ANXA5, and heat shock 70 kDa protein 5 (HSPA5)] showed branch-site positive selection, whereas for seven genes [ANXA2, lactotransferrin (LTF), OVGP1, PLG, S100 calcium-binding protein A11 (S100A11), Sperm adhesion molecule 1 (SPAM1), and osteopontin (SPP1)] branch-site model and model-site positive selection were observed. These results strongly suggest that genes encoding oviductal proteins that are known to be important for gamete fertilization are subjected to positive selection during evolution, as numerous genes encoding proteins from mammalian seminal fluid. This suggests that such a rapid evolution may have as a consequence that two isolated populations become separate species more rapidly.

## Introduction

The oviduct, also called the Fallopian tube in human, is a tubular organ that plays an important role in mammalian fertilization. The oviduct is the location where fertilization takes place in amniotes. It is involved in gamete transportation and maturation, sperm capacitation, and it provides the appropriate microenvironment for the early embryo development (Coy et al. 2012a; Hunter 1998, 2012). The oviductal fluid (OF) is formed by the secretion of the epithelial cells and also results from a transudate of the blood plasma, and contains glycosaminoglycans, metabolites, amino acids, inorganic salts, and proteins (Aviles et al. 2010; Leese et al. 2008, 2001).

The proteome of the OF has been studied in several mammalian species, such as cow (Lamy et al. 2016), ewe (Soleilhavoup et al. 2016), mare (Smits et al. 2017), pig (Georgiou et al. 2005; Mondejar et al. 2012), and rabbit (Yu et al. 2016). It has been demonstrated that the oviductal proteome changes with the different stages of the oestrous cycle (Lamy et al. 2016; Soleilhavoup et al. 2016), the presence of gametes (Georgiou et al. 2005) or the presence of embryos (Smits et al. 2017). The proteome of the oviduct epithelial cells has also been studied in humans (Wang et al. 2016) and pigs (Seytanoglu et al. 2008).

We have chosen to study the evolution of 16 genes that have been demonstrated to be crucial for the biological function of oviduct. Oviductal proteins, like oviductin (OVGP1), annexins (ANXA), heat shock proteins (HSP), deleted in malignant brain tumor (DMBT1), or osteopontin (SPP1) among others have been extensively studied (Coy and Yanagimachi 2015). These proteins are involved in different steps that are crucial for fertilization. For instance, annexins and DMBT1 participate in the binding between the sperm and the oviduct (Ignotz et al. 2007; Teijeiro et al. 2011). Progestagen-associated endometrial protein (PAEP) also known as glycodelin (Gd), lactotransferrin (LTF), heat shock 70 kDa protein 5 (HSPA5), OVGP1, prostaglandin D2 synthase (PTGDS), plasminogen (PLG), and SPP1 participate in the zona pellucida–sperm interaction controlling polyspermy (Algarra et al. 2016; Chiu et al. 2007; Coy et al. 2008, 2012b; Goncalves et al. 2008a; Hao et al. 2006; Lachance et al. 2007; Marin-Briggiler et al. 2010; Mondejar et al. 2013; Zumoffen et al. 2013). Heat shock 70 kDa protein 8 (HSPA8) has a beneficial effect on sperm viability (Elliott et al. 2009; Lloyd et al. 2009; Moein-Vaziri et al. 2014). Natriuretic peptide A (NPPA) and glycodelin-A (GdA) induce the acrosome reaction (Chiu et al. 2010; Zhang et al. 2006). Sperm adhesion molecule 1 (SPAM1) plays a role in the dispersion of the cumulus cells (Griffiths et al. 2008) and S100 calcium-binding protein A11 (S100A11) plays a role in sperm selection through its action on cumulus cells (Hanaue et al. 2011).

It is well known that genes related to reproduction evolve faster than genes expressed in most other tissues (Singh and Kulathinal 2000; Swanson and Vacquier 2002; Turner and Hoekstra 2008; Vacquier 1998); genes subjected to rapid evolution show a big percentage of amino acids substitution between species. For example, some genes involved in oocyte–sperm interaction were highlighted as ZP2, ZP3, or acrosine (Makalowski and Boguski 1998). Rapid evolution can also be associated to a loss of functionality, sometimes leading to pseudogenization (Bodmer and Ashburner 1984; Hellberg and Vacquier 1999; Li et al. 1981), or it can be related to an adaptive evolution induced by natural selection (Swanson and Vacquier 2002). This rapid evolution can be species-specific and it could even play a role in speciation, as it has been described for instance for CD9 in the oocyte and IZUMO1 in the sperm (Claw et al. 2014).

Several studies have been focused on the positive selection of the seminal plasma proteins in Drosophila, in rodents, and in primates (Meslin et al. 2015). However, to our knowledge, there are no studies focusing on the evolution and an eventual positive selection of the genes encoding oviductal proteins in any mammalian species. For this reason, the aim of this work was to study the evolution of these genes in mammals.

## Materials and Methods

### Phylogenetic and Syntenic Analyses

Sixteen oviductal proteins (ANXA1, ANXA2, ANXA4, ANXA5, DMBT1, PAEP, HSPA5, HSPA8, LTF, NPPA, OVGP1, PLG, PTGDS, SPAM1, SPP1, and S100A11), described in the literature as important for fertilization, were chosen for the analysis. On this study, the genomes of 16 placental mammal species were sampled; different species were chosen due to the availability of their genome and to include species from all the phylogenetic tree of mammals (*Ailuropoda melanoleuca, Bos taurus, Canis lupus familiaris, Equus caballus, Felis catus, Homo sapiens, Myotis lucifugus, Mus musculus, Oryctolagus cuniculus, Ovis aries, Pongo abelii, Pteropus vampyrus, Rattus norvegicus, Sus scrofa, Tarsius syrichta*, and *Tursiops truncatus*). The version of Ensembl used to perform the analyses was Ensembl 87: Dec 2016, and the following versions of genomes were used: cat (Felis_catus_6.2), cow (UMD3.1), horse (EquCab2), human (GRCh38.p7), dog (CanFam3.1), dolphin (turTru1), megabat (pteVam1), microbat (Myoluc2.0), mouse (GRCm38.p5), orangutan (PPYG2), panda (ailMel1), pig (Sscrofa10.2), rabbit (OryCun2.0), rat (Rnor_6.0), sheep (Oar_v3.1), and tarsier (tarSyr1).

For all identified genes, we used the PhyleasProg web server v3.1 (http://phyleasprog.inra.fr/) (Busset et al. 2011) to study the evolution of the corresponding Ensembl protein ID (Table 1), retrieved from the Ensembl database. We carefully examined all reconstructed phylogenetic trees before the interpretation of the results, that we eventually corrected by synteny analysis as previously described (Tian et al. 2009), so that calculations were performed with correct orthologs.

**Table 1:** List of the 16 proteins studied. Human sequences of the proteins were used for analyses

Protein	Abbreviation	Protein accession number
Annexin 1	ANXA1	ENSP00000366109
Annexin 2	ANXA2	ENSP00000346032
Annexin 4	ANXA4	ENSP00000377833
Annexin 5	ANXA5	ENSP00000296511
Deleted in malignant brain tumor	DMBT1	ENSP00000357905
Heat shock 70 kDa protein 5	HSPA5	ENSP00000324173
Heat shock 70 kDa protein 8	HSPA8	ENSP00000432083
Lactotransferrin	LTF	ENSP00000231751
Natriuretic peptide A	NPPA	ENSP00000365663
Oviductin	OVGP1	ENSP00000358747
Progestagen-associated endometrial protein	PAEP	ENSP00000417898
Plasminogen	PLG	ENSP00000308938
Prostaglandin D2 synthase	PTGDS	ENSP00000360687
Sperm adhesion molecule 1	SPAM1	ENSP00000345849
Osteopontin	SPP1	ENSP00000237623
S100 calcium-binding protein A11	S100A11	ENSP00000271638

### Identification of Pseudogenes—Inference of Positive Selection

We systematically performed the search for pseudogenes by using tBlastn in the studied genomes for genes for which one of the species of interest presented no ortholog. As previously described for seminal plasma genes, this methodology allowed us to test the hypothesis that evolution of oviductal genes in mammals is characterized by a gene loss pattern (Meslin et al. 2011, 2015; Tian et al. 2009). The pseudogene status was inferred in a genome if we found a premature stop codon or an indel in the sequence identified by the similarity search in the syntenic locus in comparison with the other species of interest. In the cases of absent genes without pseudogene observed in the synteny locus, we could only make the hypothesis that the gene has been lost.

The PhyleasProg web server used the CODEML application from the PAML package version 4.4 to investigate selective pressure (Yang 2007). This allows the calculation of the ratio d*N*/d*S* which varied across codons and the estimation of the probability for each codon to be under positive selection. We used MUSCLE and PAL2NAL softwares to obtain the alignments (Thompson et al. 1994; Suyama et al. 2006). We systematically and carefully examined multiple alignments to avoid false-positive results. In particular, we did not consider amino acids that were at the boundary of the alignments. We also systematically eliminated genes that presented sequence errors in Ensembl according to other databases such as RefSeq in NCBI, in particular for ANXA1 and PLG in rabbit; OVG1 in horse, megabat, and mouse; and SPAM1 in megabat.

Site Models implemented in PAML were used to evaluate if the intensity of selective pressure varies among sites in the sequences studied (Nielsen and Yang 1998). This allowed the estimation of *ω* ratio to vary among sites (Nielsen and Yang 1998; Yang 2000). Five models and three comparisons are used in PhyleasProg: M1a (0 < *ω*_0_ < 1 and *ω*_1_ = 1) versus M2a (0 < *ω*_0_ < 1, *ω*_1_ = 1 and *ω*_2_ > 1) (Wong et al. 2004; Yang et al. 2005), M7 (0 < *ω* < 1) versus M8 (0 < *ω* < 1 and *ω*_S_ > 1) (Yang 2000), and M8 versus M8a (0 < *ω* < 1 and *ω*_S_ = 1) (Swanson et al. 2003). We used likelihood ratio tests (LRTs) to compare the log likelihood values (Nielsen and Yang 1998). We used Bayes Empirical Bayes (BEB) method (Yang et al. 2005) implemented in PAML to estimate posterior probabilities of selection on each codon. Probabilities higher than 0.95 were considered significant.

Studying whether one or more species have undergone selection pressure on some of their genes during evolution has led us to use the Phyleasprog tool using branch-site models of PAML (Yang and Nielsen 2002; Zhang et al. 2005). These models can detect positive selection episodes on specific branches of the phylogenetic tree, designated by the user. Two types of branches are defined in these models, the so-called foreground branches for which positive selection will be allowed, (defined a priori by the user), and the so-called background branches, for which the d*N*/d*S* ratio is allowed to vary only between 0 and 1. Two models are used, one model allowing positive selection, called alternative model, and the other not allowing it, called null model. In the latter model, the d*N*/d*S* ratio *ω*_2_ is fixed to 1. For the alternative model, three classes are defined: *ω*_0_: d*N*/d*S* < 1, *ω*_1_: d*N*/d*S* = 1, and *ω*_2_: d*N*/d*S* ≥ 1. As for the site model, LRT (Nielsen and Yang 1998) and BEB (Yang et al. 2005) were used.

We tested simultaneously each branch of each phylogenetic tree for positive selection. The *q* value was used to control the statistical evidence associated with each branch tested because multiple-hypothesis tests were performed. Similar to the *p* value, we used the *q* value to measure the significance in terms of false discovery rate rather than false-positive rate. We used R package QVALUE to compute the *q* values (Storey and Tibshirani 2003). We considered positive selection on the foreground branch to be significant with a threshold of *q* < 10% of false positives. After validation of the branch with the *q* value, we only considered sites with posterior probabilities of Bayes Empirical Bayes analyses superior to 95% or 99%. We discarded from subsequent analysis datasets with less than ten sequences, which is the minimum threshold required to obtain significant results, with excessively divergent sequences, or with sequences of genes for which annotations are not reliable.

## Results

### Identification of Pseudogenes and Paralogs

**Fig. 1:**
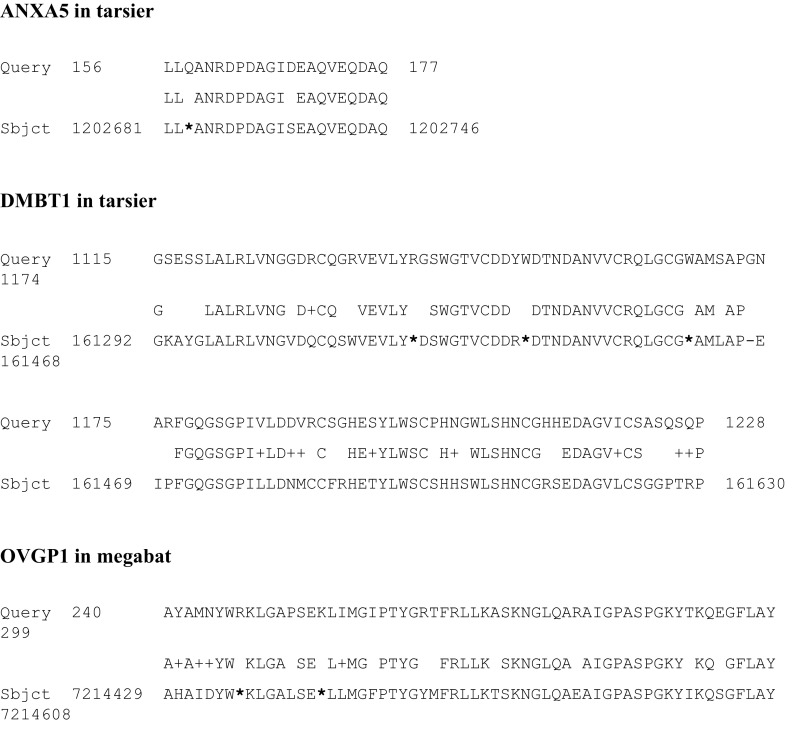
Identification of marks of pseudogenes. A tBlastn analysis allowed the identification of exons presenting STOP codons (*) within tarsier ANXA5, tarsier DMBT1, and megabat OVGP1. For each alignment, the upper number corresponds to the amino acid position and the lower number to the genomic position of nucleotides on the corresponding chromosome

**Fig. 2:**
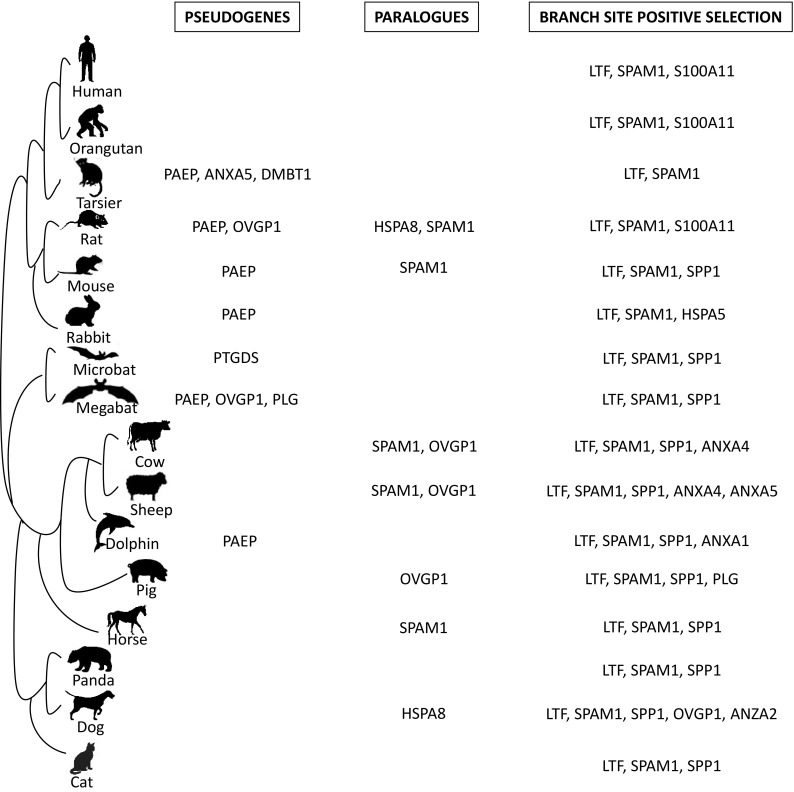
Proteins present in oviductal fluids and phylogenetic results showing gene loss, gene duplication, and positive selection. The list of oviductal proteins involved in the different processes of sperm storage and gamete fertilization in different mammals was established in the “Material and Methods” section

The present search for pseudogenes showed that ANXA5 and DMBT1 were lost in tarsier; OVGP1 in megabat; and probably PAEP in tarsier, mouse, rat, rabbit, dolphin, and megabat; PTGDS in microbat; and PLG in megabat (examples of traces of pseudogenes in Fig. 1; pseudogenes and species affected in Fig. 2).

Paralogues (*n*) were found for the next genes: OVGP1 in sheep (1), cow (2), and pig (1); HSPA8 in rat (3) and dog (1); and SPAM1 in mouse (1), rat (1), horse (1), cow (1), and sheep (1) (Fig. 2).

### Inference of Positive Selection

Five genes did not show positive selection: DMBT1, HSPA8, NPPA, PAEP, and PTGDS. Seven genes showed site model positive selection: ANXA2, LTF, OVGP1, PLG, S100A11, SPAM1, and SPP1; and 11 genes showed branch model positive selection: ANXA1, ANXA2, ANXA4, ANXA5, HSPA5, LTF, OVGP1, PLG, S100A11, SPAM1, and SPP1.

Site model positive selection was found for OVGP1, PLG, S100A11, and SPP1 with the M8a versus M8 comparison, whereas LTF and SPAM1, LRTs for both comparisons (M1 vs. M2, M8a vs. M8) were significant (*p* < 0.001) for the dataset. However, as these genes evolve particularly rapidly, the protein sequences are very divergent between species, and the identification of amino acids that are under positive selection was very difficult. So, we decided not to include this part of result because they are not confident.

The branch model showed positive selection for ANXA1, ANXA 2, ANXA4, ANXA5, HSPA5, LTF, OVGP1, PLG, S100A11, SPAM1, and SPP1 genes. The comparison between site models of PAML detects positive selection only if the *ω* ratio averaged over all branches on the tree is greater than 1, but it can also be expected positive selection to affect only a few amino acid residues along particular lineages. So, the branch-site models were used (Yang and Nielsen 2002), which are dedicated to detect such signals of local episodic positive selection, to determine whether different species have undergone selective pressure. Sixteen species were tested as foreground branch with branch-site models of PAML, branches that were tested are indicated in Fig. 2.

Significant LRTs (with at least *p* < 0.05) were found for ANXA1 in dolphin; ANXA 2 and OVGP1 in dog; ANXA4 in ruminants (cow and sheep); ANXA5 in sheep; HSPA5 in rabbit; PLG in pig; S100A11 in human, orangutan, and rat; SPP1 in microbat, megabat, horse, dog, panda, cat, pig, dolphin, cow, sheep, and mouse; and LTF and SPAM1 in all the studied species suggesting that orthologs of these genes might have been subjected to positive selection in these species (Table 2).

**Table 2:** Parameter estimates and likelihood scores for branch-site evolutionary models

Species	Model	*ɭ* ^a^	Estimates of parameters^b^	2∆*l*^c^	Positively selected sites (BEB)^d^
ANXA1—parameter estimates and likelihood scores for branch-site models for species					
Dolphin	Null	− 2590.515664	*ρ*_0_ = 0.557, (*ρ*_1_ = 0.123), *ω*_0_ = 0.046, (*ω*_1_ = 1)	3.915*	Not allowed
	Alternative	− 2588.557995	*ρ*_0_ = 0.750, *ρ*_1_ = 0.160, (*ρ*_2_ = 0.087), *ω*_0_ = 0.049, (*ω*_1_ = 1), *ω*_2_ = 7.275		1 Site *p* > 95%: 5N
ANXA2—parameter estimates and likelihood scores for branch-site models for species					
Dog	Null	− 3141.701658	*ρ*_0_ = 0.312, (*ρ*_1_ = 0.007), *ω*_0_ = 0.071, (*ω*_1_ = 1)	6.423*	Not allowed
	Alternative	− 3138.489876	*ρ*_0_ = 0.535, *ρ*_1_ = 0.011, (*ρ*_2_ = 0.072), *ω*_0_ = 0.072, (*ω*_1_ = 1), *ω*_2_ = 2.726		8 Sites *p* > 99%: 32A, 108A109E, 110S, 111L, 172I, 249H, 281Q. 16 sites *p* > 95%: 80T, 143P, 159T, 162P, 184W, 190W, 209T, 235G, 242G, 246Q, 282I
ANXA4—parameter estimates and likelihood scores for branch-site models for species					
Ruminants (cow, sheep)	Null	− 2537.951438	*ρ*_0_ = 0.886, (*ρ*_1_ = 0.074), *ω*_0_ = 0.034, (*ω*_1_ = 1)	4.451*	Not allowed
	Alternative	− 2535.725639	*ρ*_0_ = 0.915, *ρ*_1_ = 0.074, (*ρ*_2_ = 0.009), *ω*_0_ = 0.034, (*ω*_1_ = 1), *ω*_2_ = 18.872		Cow: 1 site *p* > 95%: 216A Sheep: 1 site *p* > 95%: 217T
ANXA5—parameter estimates and likelihood scores for branch-site models for species					
Sheep	Null	− 2580.297972	*ρ*_0_ = 0.734, (*ρ*_1_ = 0.063), *ω*_0_ = 0.018, (*ω*_1_ = 1)	3.937*	Not allowed
	Alternative	− 2578.329334	*ρ*_0_ = 0.903, *ρ*_1_ = 0.077, (*ρ*_2_ = 0.018), *ω*_0_ = 0.018, (*ω*_1_ = 1), *ω*_2_ = 29.298		1 Site *p* > 95%: 309R
HSPA5—parameter estimates and likelihood scores for branch-site models for species					
Rabbit	Null	− 5737.704417	*ρ*_0_ = 0.962, (*ρ*_1_ = 0.017), *ω*_0_ = 0.001, (*ω*_1_ = 1)	10.735**	Not allowed
	Alternative	− 5732.336651	*ρ*_0_ = 0.975, *ρ*_1_ = 0.016, (*ρ*_2_ = 0.007), *ω*_0_ = 0.001, (*ω*_1_ = 1), *ω*_2_ = 998.999		1 Site *p* > 95%: 329S
LTF—parameter estimates and likelihood scores for branch-site models for species					
Mammals	Null	− 12709.754534	*ρ*_0_ = 0.632, (*ρ*_1_ = 0.169), *ω*_0_ = 0.108, (*ω*_1_ = 1)	90.879***	Not allowed
	Alternative	− 12664.314683	*ρ*_0_ = 0.605, *ρ*_1_ = 0.357, (*ρ*_2_ = 0.036), *ω*_0_ = 0.106, (*ω*_1_ = 1), *ω*_2_ = 3.836		Rabbit: 4 sites *p* > 99%: 32P, 39S, 62L, 642A; 3 sites *p* > 95%: 36A, 61A, 183Q Mouse: 4 sites *p* > 99%: 31N, 38L, 61R, 641Q; 3 sites *p* > 95%: 35E, 60T, 182D Rat: 4 sites *p* > 99%: 32R, 39F, 62P, 641C; 3 sites *p* > 95%: 36Q, 61M, 182S Tarsier: 4 sites *p* > 99%: 32N, 39Y, 62R, 534R; 3 sites *p* > 95%: 36T, 61V, 183R Orangutan: 4 sites *p* > 99%: 34Q, 41F, 64T, 646S; 3 sites *p* > 95%: 38T, 63P, 185G Human: 4 sites *p* > 99%: 32Q, 39F, 62I, 644S; 3 sites *p* > 95%: 36T, 61P, 183G Dog: 4 sites *p* > 99%: 32K, 39S, 62Q, 642T; 3 sites *p* > 95%: 36K, 61R, 183K Panda: 4 sites *p* > 99%: 32K, 39S, 62Q, 640A; 3 sites *p* > 95%: 36A, 61H, 183R Cat: 4 sites *p* > 99%: 32Q, 39T, 62Q, 645D; 3 sites *p* > 95%: 36T, 61R, 183K Microbat: 4 sites *p* > 99%: 32P, 39F, 62T, 644R; 3 sites *p* > 95%: 36T, 61Y, 183R Megabat: 4 sites *p* > 99%: 32K, 39S, 62K, 641S; 3 sites *p* > 95%: 36A, 61H, 183T Horse: 4 sites *p* > 99%: 32P, 39A, 62F, 642P; 3 sites *p* > 95%: 36A, 61S, 183K Pig: 4 sites *p* > 99%: 32T, 39R, 60T, 638K; 3 sites *p* > 95%: 36S, 59P, 178N Dolphin: 4 sites *p* > 99%: 32L, 39Y, 62F, 627K; 3 sites *p* > 95%: 36L, 61R, 183K Cow: 4 sites *p* > 99%: 32Q, 39R, 62L, 642K; 3 sites *p* > 95%: 36F, 61A, 183Q Sheep: 4 sites *p* > 99%: 32P, 39Y, 62L, 642K; 3 sites *p* > 95%: 36S, 61A, 183K
Artiodactyls (pig, dolphin, cow, sheep)	Null	− 12706.881324	*ρ*_0_ = 0.608, (*ρ*_1_ = 0.337), *ω*_0_ = 0.104, (*ω*_1_ = 1)	10.855**	Not allowed
	Alternative	− 12701.453814	*ρ*_0_ = 0.622, *ρ*_1_ = 0.354, (*ρ*_2_ = 0.022), *ω*_0_ = 0.108, (*ω*_1_ = 1), *ω*_2_ = 6.298		Dolphin: 1 site *p* > 95%: 583V Sheep: 1 site *p* > 95%: 597T
Pig	Null	− 12707.737211	*ρ*_0_ = 0.587, (*ρ*_1_ = 0.333), *ω*_0_ = 0.106, (*ω*_1_ = 1)	18.188***	Not allowed
	Alternative	− 12698.643012	*ρ*_0_ = 0.621, *ρ*_1_ = 0.356, (*ρ*_2_ = ), *ω*_0_ = 0.022 0.107, (*ω*_1_ = 1), *ω*_2_ = 999.000		1 Site *p* > 95%: 49N
Dolphin	Null	− 12709.096501	*ρ*_0_ = 0.594, (*ρ*_1_ = 0.338), *ω*_0_ = 0.108, (*ω*_1_ = 1)	7.501**	Not allowed
	Alternative	− 12705.345589	*ρ*_0_ = 0.629, *ρ*_1_ = 0.353, (*ρ*_2_ = 0.015), *ω*_0_ = 0.109, (*ω*_1_ = 1), *ω*_2_ = 16.085		1 Site *p* > 95%: 507D
Rodents (mouse, rat)	Null	− 12694.848599	*ρ*_0_ = 0.543, (*ρ*_1_ = 0.306), *ω*_0_ = 0.091, (*ω*_1_ = 1)	4.198*	Not allowed
	Alternative	− 12692.749587	*ρ*_0_ = 0.586, *ρ*_1_ = 0.327, (*ρ*_2_ = 0.085), *ω*_0_ = 0.097, (*ω*_1_ = 1), *ω*_2_ = 2.516		Mouse: 1 site *p* > 99%: 180Q Rat: 1 site *p* > 99%: 180Q
Rat	Null	− 12694.360640	*ρ*_0_ = 0.481, (*ρ*_1_ = 0.260), *ω*_0_ = 0.100, (*ω*_1_ = 1)	21.139***	Not allowed
	Alternative	− 12683.791136	*ρ*_0_ = 0.561, *ρ*_1_ = 0.300, (*ρ*_2_ = 0.136), *ω*_0_ = 0.101, (*ω*_1_ = 1), *ω*_2_ = 5.662		1 Site *p* > 99%: 79S; 5 sites *p* > 95%: 86F, 145A, 186R, 514F, 696I
Chiroptera (microbat, megabat)	Null	− 12709.210769	*ρ*_0_ = 0.614, (*ρ*_1_ = 0.350), *ω*_0_ = 0.106, (*ω*_1_ = 1)	7.019**	Not allowed
	Alternative	− 12705.700835	*ρ*_0_ = 0.630, *ρ*_1_ = 0.355, (*ρ*_2_ = 0.012), *ω*_0_ = 0.109, (*ω*_1_ = 1), *ω*_2_ = 11.651		Microbat: 1 site *p* > 95%: 12L Megabat: 1 site *p* > 95%: 12W
OVGP1—parameter estimates and likelihood scores for branch-site models for species					
Dog	Null	− 4378.052266	*ρ*_0_ = 0.519, (*ρ*_1_ = 0.239), *ω*_0_ = 0.074, (*ω*_1_ = 1)	3.908*	Not allowed
	Alternative	− 4376.097858	*ρ*_0_ = 0.621, *ρ*_1_ = 0.279, (*ρ*_2_ = 0.098), *ω*_0_ = 0.076, (*ω*_1_ = 1), *ω*_2_ = 4.454		1 Site *p* > 95%: 70T
PLG—parameter estimates and likelihood scores for branch-site models for species					
Pig	Null	− 13182.998657	*ρ*_0_ = 0.775, (*ρ*_1_ = 0.197), *ω*_0_ = 0.072, (*ω*_1_ = 1)	6.215*	Not allowed
	Alternative	− 13179.890971	*ρ*_0_ = 0.792, *ρ*_1_ = 0.199, (*ρ*_2_ = 0.006), *ω*_0_ = 0.073, (*ω*_1_ = 1), *ω*_2_ = 21.805		1 Site *p* > 95%: 620K
S100A11—parameter estimates and likelihood scores for branch-site models for species					
Primates (human, orangutan)	Null	− 1468.549296	*ρ*_0_ = 0.795, (*ρ*_1_ = 0.153), *ω*_0_ = 0.044, (*ω*_1_ = 1)	4.798*	Not allowed
	Alternative	− 1466.149841	*ρ*_0_ = 0.825, *ρ*_1_ = 0.158, (*ρ*_2_ = 0.015), *ω*_0_ = 0.044, (*ω*_1_ = 1), *ω*_2_ = 19.752		Human: 1 site *p* > 95%: 78S Orangutan: 1 site *p* > 95%: 78S
Rat	Null	− 1464.292877	*ρ*_0_ = 0.697, (*ρ*_1_ = 0.128), *ω*_0_ = 0.038, (*ω*_1_ = 1)	6.934**	Not allowed
	Alternative	− 1460.825580	*ρ*_0_ = 0.811, *ρ*_1_ = 0.150, (*ρ*_2_ = 0.036), *ω*_0_ = 0.041, (*ω*_1_ = 1), *ω*_2_ = 45.087		1 Site *p* > 95%: 8L
SPAM1—parameter estimates and likelihood scores for branch-site models for species					
Mammals	Null	− 9332.838271	*ρ*_0_ = 0.486, (*ρ*_1_ = 0.222), *ω*_0_ = 0.088, (*ω*_1_ = 1)	55.457***	Not allowed
	Alternative	− 9305.109656	*ρ*_0_ = 0.461, *ρ*_1_ = 0.470, (*ρ*_2_ = 0.067), *ω*_0_ = 0.086, (*ω*_1_ = 1), *ω*_2_ = 2.833		Rabbit: 3 sites *p* > 99%: 121Q, 131L, 403G Mouse: 3 sites *p* > 99%: 121S, 131L, 401Q Rat: 3 sites *p* > 99%: 121S, 131H, 403R Tarsier: 3 sites *p* > 99%: 122K, 132S, 403I Orangutan: 3 sites *p* > 99%: 122D, 132L, 403D Human: 3 sites *p* > 99%: 122D, 132T, 403D Microbat: 3 sites *p* > 99%: 121A, 131E, 403K Dog: 3 sites *p* > 99%: 122K, 132S, 404V Panda: 3 sites *p* > 99%: 122K, 132A, 404D Cat: 3 sites *p* > 99%: 122K, 132S, 404V Pig: 3 sites *p* > 99%: 122R, 132L, 403A Dolphin: 3 sites *p* > 99%: 122E, 132S, 404Q Cow: 3 sites *p* > 99%: 123S, 133A, 405M Sheep: 3 sites *p* > 99%: 122N, 132A, 404M Horse: 3 sites *p* > 99%: 118A, 128S, 399K
Rodents (mouse, rat, rabbit) and primates (human, orangutan, tarsier)	Null	− 9332.291017	*ρ*_0_ = 0.481, (*ρ*_1_ = 0.494), *ω*_0_ = 0.085, (*ω*_1_ = 1)	20.661***	Not allowed
	Alternative	− 9321.960143	*ρ*_0_ = 0.474, *ρ*_1_ = 0.485, (*ρ*_2_ = 0.039), *ω*_0_ = 0.087, (*ω*_1_ = 1), *ω*_2_ = 4.121		Rabbit: 1 site *p* > 95%: 131L Mouse: 1 site *p* > 95%: 131L Rat: 1 site *p* > 95%: 131H Tarsier: 1 site *p* > 95%: 132S Orangutan: 1 site *p* > 95%: 132L Human: 1 site *p* > 95%: 132T
Microbat	Null	− 9328.404724	*ρ*_0_ = 0.415, (*ρ*_1_ = 0.432), *ω*_0_ = 0.077, (*ω*_1_ = 1)	21.585***	Not allowed
	Alternative	− 9317.611891	*ρ*_0_ = 0.443, *ρ*_1_ = 0.456, (*ρ*_2_ = 0.100), *ω*_0_ = 0.081, (*ω*_1_ = 1), *ω*_2_ = 11.322		1 site *p* > 99%: 374L ; 2 sites *p* > 95%: 392A, 423M
Pig	Null	− 9328.072812	*ρ*_0_ = 0.400, (*ρ*_1_ = 0.422), *ω*_0_ = 0.078, (*ω*_1_ = 1)	10.202**	Not allowed
	Alternative	− 9322.971449	*ρ*_0_ = 0.468, *ρ*_1_ = 0.491, (*ρ*_2_ = 0.039), *ω*_0_ = 0.081, (*ω*_1_ = 1), *ω*_2_ = 13.407		1 site *p* > 95%: 77T ; 1 site *p* > 99%: 413N
SPP1—parameter estimates and likelihood scores for branch-site models for species					
Microbat, megabat, horse, dog, panda, cat, pig, dolphin, cow, sheep	Null	− 3405.427792	*ρ*_0_ = 0.521, (*ρ*_1_ = 0.158), *ω*_0_ = 0.180, (*ω*_1_ = 1)	4.387*	Not allowed
	Alternative	− 3403.234241	*ρ*_0_ = 0.611, *ρ*_1_ = 0.142, (*ρ*_2_ = 0.245), *ω*_0_ = 0.226, (*ω*_1_ = 1), *ω*_2_ = 1.603		Microbat: 3 sites *p* > 95%: 291H, 299P Megabat: 3 sites *p* > 95%: 108S, 254R, 262P Horse: 3 sites *p* > 95%: 107S, 280S, 288R Dog: 3 sites *p* > 95%: 104L, 244H, 252S Panda: 3 sites *p* > 95%: 104G, 266H, 274S Cat: 3 sites *p* > 95%: 104T, 266N, 274P Pig: 3 sites *p* > 95%: 107A, 271H, 279S Dolphin: 3 sites *p* > 95%: 87A, 256R, 264L Cow: 3 sites *p* > 95%: 103S, 244L, 252H Sheep: 3 sites *p* > 95%: 105S, 247L, 255H
Dog, panda, cat, pig, dolphin, cow, sheep	Null	− 3408.017517	*ρ*_0_ = 0.559, (*ρ*_1_ = 0.230), *ω*_0_ = 0.201, (*ω*_1_ = 1)	6.525*	Not allowed
	Alternative	− 3404.754608	*ρ*_0_ = 0.602, *ρ*_1_ = 0.234, (*ρ*_2_ = 0.162), *ω*_0_ = 0.221, (*ω*_1_ = 1), *ω*_2_ = 2.002		Dog: 1 site *p* > 95%: 104L Panda: 1 site *p* > 95%: 104G Cat: 1 site *p* > 95%: 104T Pig: 1 site *p* > 95%: 107A Dolphin: 1 site *p* > 95%: 87A Cow: 1 site *p* > 95%: 103S Sheep: 1 site *p* > 95%: 105S
Dog, panda, cat	Null	− 3404.586199	*ρ*_0_ = 0.443, (*ρ*_1_ = 0.204), *ω*_0_ = 0.197, (*ω*_1_ = 1)	18.383***	Not allowed
	Alternative	− 3395.394529	*ρ*_0_ = 0.556, *ρ*_1_ = 0.245, (*ρ*_2_ = 0.197), *ω*_0_ = 0.207, (*ω*_1_ = 1), *ω*_2_ = 4.297		Dog: 2 sites *p* > 99%: 104L, 240L; 2 sites *p* > 95%: 75S, 182S Panda: 2 sites *p* > 99%: 104G, 262I; 2 sites *p* > 95%: 75S, 182S Cat: 2 sites *p* > 99%: 104T, 262T; 2 sites *p* > 95%: 75A, 182S
Dog, panda	Null	− 3410.889147	*ρ*_0_ = 0.600, (*ρ*_1_ = 0.399), *ω*_0_ = 0.208, (*ω*_1_ = 1)	10.162**	Not allowed
	Alternative	− 3405.808026	*ρ*_0_ = 0.586, *ρ*_1_ = 0.393, (*ρ*_2_ = 0.018), *ω*_0_ = 0.208, (*ω*_1_ = 1), *ω*_2_ = 33.914		Dog: 1 site *p* > 95%: 104L Panda: 1 site *p* > 95%: 104G
Pig, dolphin, cow, sheep	Null	− 3410.383104	*ρ*_0_ = 0.584, (*ρ*_1_ = 0.368), *ω*_0_ = 0.204, (*ω*_1_ = 1)	8.998**	Not allowed
	Alternative	− 3405.883945	*ρ*_0_ = 0.569, *ρ*_1_ = 0.372, (*ρ*_2_ = 0.057), *ω*_0_ = 0.206, (*ω*_1_ = 1), *ω*_2_ = 4.776		Pig: 1 site *p* > 95%: 176Q Dolphin: 1 site *p* > 95%: 156Y Cow: 1 site *p* > 95%: 172S Sheep: 1 site *p* > 95%: 175S
Mouse	Null	− 3408.601339	*ρ*_0_ = 0.350, (*ρ*_1_ = 0.254), *ω*_0_ = 0.186, (*ω*_1_ = 1)	7.352**	Not allowed
	Alternative	− 3404.925140	*ρ*_0_ = 0.565, *ρ*_1_ = 0.394, (*ρ*_2_ = 0.039), *ω*_0_ = 0.195, (*ω*_1_ = 1), *ω*_2_ = 30.713		1 Site *p* > 95%: 35L

^a^Log-likelihood values

^b^ *ρ*_0_, *ρ*_1_, and *ρ*_2_ are the proportions of codons subject to purifying, neutral, and positive selection, respectively. *ω*_0_, *ω*_1_, and *ω*_2_ represented d*N*/d*S* for each class (purifying, neutral, and positive selection, respectively)

^c^*Significant at *p* < 0.05 threshold; **significant at *p* < 0.01 threshold; ***significant at *p* < 0.001 threshold

^d^Bayes Empirical Bayes inference of amino acid sites under positive selection

## Discussion

Previous studies have identified numerous proteins in the OF from several mammalian species (Georgiou et al. 2005; Lamy et al. 2016; Mondejar et al. 2012; Smits et al. 2017; Soleilhavoup et al. 2016; Yu et al. 2016). Their expression changes with stages of the oestrous cycle (Lamy et al. 2016; Soleilhavoup et al. 2016), presence of gametes (Georgiou et al. 2005), or presence of embryos (Smits et al. 2017). Some of these proteins are involved in crucial steps for fertilization, such as binding between sperm and oviduct (Ignotz et al. 2007; Teijeiro et al. 2011), zona pellucida–sperm interaction (Algarra et al. 2016; Coy et al. 2012a; Goncalves et al. 2008a; Hao et al. 2006; Lachance et al. 2007; Mondejar et al. 2013; Zumoffen et al. 2013), sperm viability (Elliott et al. 2009; Lloyd et al. 2009; Moein-Vaziri et al. 2014), acrosome reaction (Chiu et al. 2010; Zhang et al. 2006), dispersion of cumulus cells (Griffiths et al. 2008), and sperm selection (Hanaue et al. 2011). The present work suggests that the high diversity of proteins present in the OF of mammals is associated with a species-specific evolutionary pattern of the corresponding genes by fairly frequent pseudogenization. Gene death is usually caused by introducing any changes that lead to a premature stop codon. Now, with the availability of complete animal genome sequences, it is possible to test hypotheses concerning gene death by searching for the trace of a pseudogene.

Pseudogenization has been previously demonstrated for OVGP1 in the rat (Tian et al. 2009). Our study shows that OVGP1 is lost in megabat. OVGP1, also known as oviductin, has been shown to play a role in the fertilization process in goat (Pradeep et al. 2011), pig (McCauley et al. 2003), cow (Martus et al. 1998), buffalo (Choudhary et al. 2017), hamster (Saccary et al. 2013; Schmidt et al. 1997; Yang et al. 2015), and human (O’Day-Bowman et al. 1996; Zhao et al. 2016): it attaches to the zona pellucida and the spermatozoa, and improves in vitro fertilization and embryo development. Interestingly, targeted invalidation of Ovgp1 in the mouse is without any consequence in female reproduction (Araki et al. 2003), implying that at least in murine species, Ovgp1 is not essential for fertility. Gene loss by pseudogenization has already been reported for genes encoding seminal fluid proteins, such as for prostate-specific transglutaminase (TGM4) genes, and semenogelines (SEM1 and SEM2) in gorillas (Carnahan and Jensen-Seaman 2008), as well as TGM4 in pigs (Meslin et al. 2015).

Our study shows that PAEP is lost in tarsier, rat, mouse, rabbit, megabat, and dolphin. PAEP, also named glycodelin-A, interacts with fucosyltransferase on human sperm plasma membrane thus inhibiting spermatozoa-zona pellucida binding (Chiu et al. 2007). Moreover, pre-treatment of spermatozoa with glycodelin-A enhances zona pellucida-induced calcium influx and zona pellucida-induced acrosome reaction (Chiu et al. 2010). Thus, PAEP is involved in sperm selection and preparation for fertilization. In tarsier, rat, mouse, rabbit, megabat, and dolphin, this role may not be essential, or may be assumed by other proteins.

Two other proteins have been lost in tarsier: ANXA5 and DMBT1. ANXA5 is a candidate for the bovine oviductal epithelium sperm receptors, it binds with high affinity to heparin and related glycosaminoglycans and antibodies to this protein block sperm–oviduct binding (Talevi and Gualtieri 2010). However, since other annexins, such as ANXA1, ANXA2, and ANXA4 are also candidates for the oviductal epithelium sperm receptors, the loss of ANXA5 may be replaced by other annexins. DMBT1 has been shown to be involved in the mechanism of fertilization in equine and porcine species (Ambruosi et al. 2013): pre-incubation of oocytes with DMBT1 induces an increase in in vitro fertilization rate and an interaction between DMBT1 and spermatozoa has been shown using surface plasmon resonance studies. Moreover, DMBT1 has been proposed to be implicated in sperm selection in the oviduct through acrosome alteration and suppression of motility (Teijeiro and Marini 2012). As previously mentioned for ANXA5, in tarsier, other candidates for sperm binding and selection are probably involved.

Finally, our study shows that PTGDS and PLG are lost in microbat and megabat, respectively. In bovine, prostaglandin D2 synthase (PTGDS) is involved in sperm binding to the ZP, in vitro fertilization and embryonic development, reaction with both oocytes and spermatozoa (Goncalves et al. 2008a, b). In swine, plasminogen (PLG) regulates sperm entry into the oocyte: sperm binding to oocytes triggers the releasing of plasminogen activators and the generated plasmin causes supernumerary spermatozoa detachment from the zona pellucida (Coy et al. 2012b). Since these proteins are lost in microbat and megabat, some other proteins are probably involved in this regulation.

Overall, this study revealed that the evolution of mammals was accompanied by the progressive loss of genes coding for oviductal proteins involved in the process of fertilization. The significance of such loss remains to be further investigated. It may be related, at least for bats, to sperm storage in the oviduct. Prolonged sperm storage in the female genital tract over winter is a common feature of reproduction in some bats, so that they can mate in the autumn but postpone fertilization until the spring (Holt 2011). Thus, oviductal proteins involved in sperm attachment to the oviduct or sperm selection within the oviduct may have evolved in a different way in these species.

In our study, gene duplication has been observed in several species: orangutan, rat, mouse, cow, sheep, pig, horse, cat, and dog. The duplication could be a preservation mechanism through which some daughter copies keep ancient functions, whereas others evolve toward new biological functions.

OVGP1 is lost in rat and megabat. Cow, sheep, and pig have two copies of the gene. Porcine OVGP1 has been shown to have an effect on the number of sperm bound to the zona pellucida, on sperm penetration rate, and polyspermy rate (Kouba et al. 2000). Sperm adhesion molecule 1 (SPAM1) is duplicated in rat, mouse, cow, sheep, and horse. SPAM1 is a hyaluronidase which can bind to sperm during capacitation and increase cumulus dispersal efficiency and ability of sperm to penetrate the cumulus of oocytes (Griffiths et al. 2008; Martin-DeLeon 2006). Prothmann et al. (2012) found that the rate of evolution of the gene encoding SPAM1 could be correlated with relative testis weight in monkeys as well as the uni-male versus multi-male breeding system. This suggests that different levels of sperm competition might account for species-specific sequence evolution of SPAM1 in these species. The evolution of gene encoding the male zonadhesin gene also may be related to the uni-male breeding system in other primates (Herlyn and Zischler 2007, 2008). In the present work, we find a positive selection of this gene in all the studied species. However, it is too speculative here to make a hypothesis concerning breeding system because we did the work on a greater diversity of mammals, with wild and domesticated animals, and very different breeding systems. However, such a question would be very interesting to investigate with a smaller and especially more homogenous group of species.

S100 calcium-binding protein A11 (S100A11) is duplicated in rat, pig, and cat. This protein plays a role in sperm selection through its action on cumulus cells (Hanaue et al. 2011). Annexin A2 (ANXA2) is duplicated in sheep and dog. ANXA2 has been shown to be present at the apical surface of the oviductal epithelial cells in sow and it is the main sperm binding isoform in pig (Teijeiro et al. 2009). Other annexins, such as ANXA1, ANXA4, and ANXA5 are other candidates for the oviductal epithelium sperm receptors (Talevi and Gualtieri 2010). Finally, HSPA8 is duplicated in orangutan, rat, sheep, and pig. HSPA8 is involved in maintenance of bovine, ovine, and porcine sperm survival in the oviduct (Elliott et al. 2009; Lloyd et al. 2009). Overall, all the proteins that are duplicated are involved in sperm selection and competence to reach the oocyte.

In our study, we have observed branch-site positive selection in all the species that we have studied.

SPAM1 showed positive selection in all the species analyzed: human, orangutan, tarsier, rat, mouse, rabbit, microbat, megabat, cow, sheep, dolphin, pig, horse, cat, dog, and panda. Moreover, SPAM1 has not been lost in the sampled species, but was duplicated in rat, mouse, cow, sheep, and horse. Since it is involved in the ability of sperm to penetrate the cumulus of oocytes (Griffiths et al. 2008; Martin-DeLeon 2006), this could highlight its species-specific role in gamete recognition.

Lactotransferrin (LTF) also showed positive selection in all the species analyzed. Moreover, LTF has neither been lost nor duplicated in the sampled species. LTF has been shown to interact with spermatozoa and oocytes and to modulate gamete interaction (Zumoffen et al. 2013). This rapid evolution may contribute to the diversity of mating systems and may explain part of the loss in interspecific fecundity after speciation (Meslin et al. 2015).

Osteopontin (SPP1) showed positive selection in many species: mouse, microbat, megabat, cow, sheep, dolphin, pig, horse, cat, dog, and panda. Moreover, SPP1 has neither been lost nor duplicated in the sampled species. SPP1 has been shown to improve bovine sperm capacitation (Monaco et al. 2009) and in vitro development of porcine embryos (Hao et al. 2008). Moreover, osteopontin plays an important role in the regulation of pig polyspermic fertilization; it decreases polyspermy and increases fertilization efficiency during IVF (Hao et al. 2006).

Annexins showed positive selection in cow (ANXA4), sheep (ANXA4 and ANXA5), dolphin (ANXA1), and dog (ANXA2). Several annexins, such as ANXA1, ANXA2, ANXA4, and ANXA5 are involved in oviductal epithelium sperm receptors. Their rapid evolution may also contribute to the loss in interspecific fecundity.

S100A11, also involved in the interaction between sperm and cumulus cells, showed positive selection only in human, orangutan and rat, and gene duplication in rat, pig and cat.

Heat shock 70 kDa protein 5 (HSPA5) showed positive selection only in the rabbit. It has neither been lost nor duplicated in the sampled species, contrary to HSPA8, which was duplicated in orangutan, rat, sheep, and pig. Heat shock proteins A5 and A8 play a role in the modulation of boar sperm function in the oviductal reservoir (Yeste et al. 2014).

Plasminogen (PLG) showed positive selection only in the pig. In porcine, PLG regulates sperm entry into the oocyte (Coy et al. 2012b). Its rapid evolution could highlight the species-specific gamete recognition.

OVGP1 showed positive selection only in the dog. It is lost in rat and megabat, and duplicated in cow, sheep, and pig. It has been shown to have an effect on the number of sperm bound to the zona pellucida, on sperm penetration rate, and polyspermy rate (Kouba et al. 2000).

## Conclusion

Overall, the present work highlights the evolution of genes encoding proteins involved in gamete transportation and maturation, sperm capacitation, and early embryo development. These genes are subjected to a particularly rapid evolution by duplication as well as species-specific positive selection, suggesting a diversification of their functions and of their role in the different mammalian species, with possible impact on the process of speciation, as proposed for the evolution of genes encoding proteins of seminal fluid of different species (Meslin et al. 2015). Moreover, several previous works have suggested that genes encoding proteins involved in sperm–egg interactions might undergo co-evolution and will also have correlated evolutionary rates due to compensatory changes on both the sperm and egg (Claw et al. 2014). In the present paper and one previous one (Meslin et al. 2015), one could hypothesize such a kind of co-evolution for proteins of seminal fluid and proteins of oviductal fluid.
